# Unseen and Invisible? Issues of Recognition for Parents With Intellectual Disabilities Accessing Social Work and Social Care Services for Adults in England

**DOI:** 10.1111/jar.70075

**Published:** 2025-08-03

**Authors:** Beth Tarleton, Gillian MacIntyre, Danielle Turney, Rhian Fawcett

**Affiliations:** ^1^ University of Bristol Bristol UK; ^2^ University of Strathclyde Glasgow UK; ^3^ Queens University Belfast Belfast UK

**Keywords:** Care Act, parents with intellectual disabilities, parents with learning difficulties, parents with learning disabilities, support, thresholds

## Abstract

**Background:**

Parents in England with an intellectual disability may be eligible for support with parenting from Local Authority's Adult services under the Care Act (2014).

**Method:**

This study investigated how adult social workers support these parents through 18 interviews with managers and commissioners and focus groups with 52 social workers, analysed using thematic analysis.

**Results:**

Parents with a diagnosed ‘intellectual disability’ accessed ‘gold standard of support’ from intellectual disability teams. Parents with a milder or borderline intellectual disability accessed support from the ‘general’ team if they had two eligible needs under the Care Act. There were inclusive and more restrictive approaches, related to a lack of resources and social worker knowledge and skills, to the recognition of two eligible needs.

**Conclusions:**

Parents with milder intellectual disabilities are rendered invisible to services due to not having a ‘label’ or ‘obvious’ eligibility for support under the Care Act.

**Summary**

- Parents with a diagnosed intellectual disability usually get support from their Local Authority's specialist intellectual disability team, while parents with a milder disability may get support from the general adults' team.
- To get support from the general adults' team, parents need to consent to a Care Act (2014) assessment and have two eligible needs. During the assessment, some social workers took an inclusive position, ensuring they identified two support needs, while others were more restrictive, limiting access to services.

## 1 Introduction

Research has shown that with the right support, many parents with intellectual disabilities can provide appropriate care for their children, allowing families to remain together (MacIntyre and Stewart 2012; Ptacek et al. 2024; Tahir and Cobigo 2024; Tarleton and Turney 2020). In England, the introduction of the Care Act in 2014 resulted in significant reforms to the legal framework for adult social care with the aim of ensuring that there was a standard way of establishing entitlement to support (Kelly 2013). The Act introduced minimum thresholds for services, which meant that parents with intellectual disabilities were potentially eligible to receive support with parenting if they were assessed as having two eligible care needs because of a ‘physical or mental impairment or illness’. Tasks associated with parenting or responsibilities an adult has for a child could be considered eligible needs under the legislation.

Previous research has discussed the negative experiences of parents with intellectual disabilities in the child protection system (Atkin et al., 2021; Rice et al., 2021), and a recent study by Burch et al., (2024) suggests that they are over‐represented within this system, with numbers much higher than previously estimated. Indeed, Burch et al. (2024) found that ‘in 34% (67) of 200 recently concluded care proceedings regarding babies across four local authorities [in England], there was reliable, mostly expert, evidence that at least one parent had intellectual disabilities or intellectual difficulties’. Parents with borderline intellectual disabilities or those who lack a formal diagnosis had not previously been included in such figures, yet they are likely to share similar experiences and challenges to parents with a diagnosed intellectual disability (Bowling and Delacruz Combs 2023) and, as a result, are likely to require similar support and accommodations. The Care Act has the potential to support this group of parents who have often been referred to as occupying a ‘no‐man's land’ between children's and adult services, where there is no access to support (Tarleton and Porter 2012; MacIntyre et al. 2019). However, while we know an increasing amount about the experiences of parents with intellectual disabilities, we know very little about how adult social workers can support this group, and this study therefore aimed to investigate how adult social workers identify parents with intellectual disabilities and how services respond to them. The following section provides a brief overview of existing literature around professional responses to parents with learning disabilities.

### 1.1 Professional Response to Parents With Intellectual Disabilities

Existing literature on professional responses to parents with intellectual disabilities does not explicitly consider the role of adult social workers but does consider other professional groups. Pytlowana and Stenfert Kroese's (2021) systematic review of other professionals' experiences of working with parents with intellectual disabilities found that working with parents with intellectual disabilities has a high emotional impact. They identified a layering of personal, professional, organisational and social values and attitudes that impact on professional practice with these parents. Professionals reported concerns regarding their own knowledge, experience, and ability to work with parents while feeling guilty that they were unable to help parents more. They reported feeling pressure to place children in alternative care arrangements permanently and at an earlier stage. They also feared the wider public response if children remained at home and were harmed, and were concerned about the availability of support for families. They highlighted difficulties with liaison between different services and the power imbalances between professionals and parents and between professionals and the court.

Other studies have highlighted the perceived negative and stereotypical expectations of parents and their parenting capacity held by some professionals. Studies by Aunos and Pacheco (2019; 2021), for example argued that intellectual disability was often considered a ‘red flag’ or safeguarding concern that suggested increased risk to children. They found that specialist intellectual disability services were likely to hold more positive views of parenting capacity, particularly when appropriate support was in place, compared to the views of non‐specialist services (See also Jones 2013; Tefre 2017; Llewellyn and McConnell 2002; Pacheco and McConnell 2017; Tilbury and Tarleton 2023). Previous studies also suggest that children's and adult services traditionally do not work well together (Tarleton et al. 2006; MacIntyre et al. 2019) and that the needs and safety of the child or children are rightly prioritised over parents' support needs. Often, work involving parents with intellectual disabilities has focused on child protection, and knowledge of how parents access support as adults is limited. This study, therefore, plays a vital role in addressing this gap by focusing specifically on the part adult social workers can play in supporting parents with intellectual disabilities.

## 2 Methods and Study Context

### 2.1 Project Design

Social work services for adults with intellectual disabilities in England are organised in two main ways. There is usually a ‘general’ team whose role is to support any adult who has eligible needs under the Care Act (2014). Some areas also have specialist intellectual disability teams (known in the United Kingdom as learning disability teams) that often include health and social care professionals, who work with adults with a recognised/diagnosed intellectual disability. This study took place across five Local Authority (LA) sites: four that had a specialist intellectual disability team – one of which was integrated with health while three worked closely with health services – and one that only had general adult services teams.

The project was guided by a professional advisory group, including academics, social workers, service managers and representatives of social work bodies and a parents' advisory group made up of parents with intellectual disabilities and advocacy staff. Both advisory groups ensured that the methods used were appropriate. The project took an innovate ecosystemic approach, previously not used in research about parents with an intellectual disability, which ‘facilitates analysis of environment and context, and knowledge translation to policy and practice’ (Furst et al. 2021, page 23) by looking at what happens at various ‘levels’ of the system.

### 2.2 Procedure

The aim of this study was to investigate how adult social workers identify parents with intellectual disabilities and how services respond to them. It did this by addressing the following research questions:

- How do service managers and commissioners expect adult social workers to work with parents with intellectual disabilities?
- In what ways do adult social workers identify and respond to, and support these parents, and how are they involved when there are concerns regarding the welfare of children?
- What knowledge and practice wisdom do adult social workers draw on when making decisions about parents with intellectual disabilities?

Ethical approval was received from the University of Bristol, School for Policy Studies Research Ethics Committee (SPSREC/22–23/280) and local ethical/research governance approval was obtained in each of the sites. Participants were recruited via a gatekeeper in each site who forwarded an email either directly to social workers or their team managers. All participants were provided with detailed information about the study via an information sheet, which specified that information would be kept confidential unless there were serious safeguarding concerns, and specific consent was asked to use quotes and archive the information shared anonymously. All participants completed an online consent form.

In order to address the research questions set out above, individual online interviews with service managers and commissioners explored how they understood their local policy context, how parents were identified in their local authority and how they believed social workers responded to parents with intellectual disabilities, including how they believed adult social workers should utilise the Care Act.

Focus group interviews were then undertaken with adults and children's social workers, which investigated how adult social workers understood their practice with parents and how this was informed by local and national policy and legislation. Children's social workers were included to allow exploration of joint working between children's and adult services, particularly when there were concerns about the welfare of a child. We aimed for three online focus groups in each area: two with social workers from adult services, one intellectual disabilities team social workers and one with general adults' team social workers, or two focus groups with general adult workers, if no specialist team existed and a focus group with children's social workers.

The focus groups utilised a vignette (MacIntyre et al. 2011 Rapaport et al. 2008) initially focusing on a couple who were presented as a potentially having a milder or borderline intellectual disability to allow for discussion as to how their needs would be viewed and responded to. Participants were also asked to discuss what local and/or national policy/law would guide them in their response. When the social workers had exhausted their discussion regarding these parents, they were asked to discuss how they would respond to the same parents if they had a diagnosed intellectual disability. If necessary, a topic guide was used to guide further discussion around local policy contexts, ‘expected’ responses to parents, the roles of the different teams and professionals and the support and training available to them about working with parents with intellectual disabilities.

The vignette, developed in conjunction with the project advisory groups, enabled social workers to contribute without discussing parents with intellectual disabilities they have worked with and allowed comparison between responses to parents with and without a diagnosed intellectual disability and between the different groups of social workers in the same local authority and across local authorities.

Online interview and focus groups were chosen to support engagement as we were aware of the rapidly changing social care landscape in England and social workers' lack of capacity to support research (Cyhlarova et al. 2020). We believed that an invitation to an in‐person focus group or interview would be off‐putting due to the extra time commitment required for hosting/travel (de Villiers et al. 2022; Salmons 2015). The focus groups ran very smoothly, with two facilitators present, and we believe that there was no loss of quality in the data as the participants and researchers were used to working online.

### 2.3 Participants

The final sample included 18 key informant interviews with social work managers and commissioners: five from intellectual disability services, seven from general teams and three from children's services and three commissioners. A total of 14 focus groups were carried out. There were five focus groups, which included 19 intellectual disability social workers. There were five focus groups, which included 22 general social workers and four focus groups with 12 children's social workers. All of the social workers (except for two student social workers) were experienced, with the majority saying they had been in their current role for at least 4 years. The majority of the managers and commissioners discussed having worked within their local authorities for most of their careers. We did not collect further demographic information from participants as this was not the central focus of our analysis. This is in line with GDPR (UK data protection) requirements, which recommend not collecting unnecessary data from participants.

### 2.4 Data Analysis

All of the interviews were analysed using thematic analysis (Braun and Clarke 2006). Each transcript was read in detail and notes made of items of interest. It was then ‘coded’, and all parts of the interview discussing the same topic were given the same label. Descriptions of responses to parents and the themes related to these were then developed by grouping the labelled comments and developing a representative theme name. This analysis was also checked by another member of the team, ensuring consistency in coding, and so that team members had an awareness of the range of material collected. The themes were recorded in a set of tables for each LA Miles, Huberman and Saldana (2019) to allow comparison between professional groups and sites.

## 3 Findings

As the aim of this study was to investigate how adult social workers identify parents with intellectual disabilities and how services respond to them, it was important to understand how parents initially accessed services. Findings suggest that there were two clear routes into services: (i) via specialist intellectual disability teams for those with a formally diagnosed intellectual disability and (ii) via general (or generic adult) team for those with a milder or borderline, undiagnosed intellectual disability who could potentially access support under the Care Act.

### 3.1 The Gold Standard of Support: Specialist Support From Intellectual Disability Teams

To access support, under the Care Act 2014, from specialist intellectual disability teams, parents had to meet what were often perceived as strict eligibility criteria related to their IQ. Three out of four LAs who had a specialist team used IQ as a key part of their ‘pathway in terms of eligibility for intellectual disability services’ (Site 4, manager 1).

Intellectual disability is about adaptive functioning, something that you're born…it has to be somebody who has an IQ of below 70 …It's not something you get. It's not something that can be cured. It's not a health condition. It is an intellectual disability, as per, you know, the World Health Organisation's classifications. (Site 2, manager 1)

Some respondents adopted this very rigid approach, which gave a sense of certainty to the threshold for access to the intellectual disability team, while acknowledging the problems with such a fixed approach. This often caused some level of discomfort:

We tend to support people… with an IQ under 70… but personally I…feel that's a really old way of doing things and a person can present very differently on a day‐to‐day basis. (Site 2, commissioner 2)

All participants agreed that support from the intellectual disability team was something of a ‘gold standard’ in support terms as it ensured access to professionals with a greater level of knowledge and understanding of intellectual disabilities who provided individualised, person‐centred support. Intellectual disability teams were often multi‐disciplinary, enabling parents to access support from a range of allied health professionals. There was a perception that these teams were better resourced and that generally parents with intellectual disabilities (including those on the borderline and those without a formal diagnosis) would be better supported by these teams.

I think the specialist teams have the specialist knowledge. And I think they have the specialist services… And to be honest with you…if I had an… intellectual disability, difficulty, whatever, I… would rather be with the ID Team. (Site 2, manager 4)

Those parents who did not meet the threshold for support from the intellectual disability team but had a milder, borderline or undiagnosed intellectual disability may have been eligible for support under the Care Act from the general adults' team.

### 3.2 Responses From General Adult Services Under the Care Act: Jack of all Trades, Master of None?

Parents who did not meet the eligibility criteria to receive support from the intellectual disability team because they had an ‘undiagnosed intellectual disability’ (Site 4, focus group 2) or a milder or borderline ‘*intellectual difficulty*’ (learning difficulties) (Site 5, manager 4) were often referred to a general adults' team:

But I think especially with people who have…intellectual difficulties, or…but they don't meet the criteria for sort of ID services…there is a need for support … and we are …very generic. We're not a speciality team…but we do tend to work with lots and lots of different people who don't fit into the criteria of the specialist teams as such. (Site 2, manager 4)

All participants within these general teams recognised their duties and responsibilities under the Care Act and understood that parents with intellectual disabilities had to have two eligible care needs to get support under the Act. However, there were two different approaches to implementation in practice, with some social workers taking a more proactive and others a more restrictive approach to assessing and identifying these needs. There was also some concern among participants that social workers from general adult teams did not have a detailed understanding of intellectual disabilities or the kinds of support needs that these parents had. This reinforced the perception that parents were ‘better off’ when they received support from the intellectual disability team.

Of the five case study sites in this study, only one did not have a specialist intellectual disability team. There was a general adults' team that covered all adults with social care needs. Staff in this area felt that they lacked specialist knowledge, while the manager stated that they were 'jack of all trades and master of none' and that the social workers were uncertain about working with people with intellectual disabilities (Site 3 Manager1).

#### 3.2.1 Consenting to a Care Act Assessment: Promoting Choice and Control

A Care Act assessment is the first step towards accessing support and requires consent from the individual to take place. This was viewed by some participants as an empowering approach which recognised parents with intellectual disabilities as adults who may need support ‘in their own right’, who could exercise choice and control. Indeed, it was vital that parents freely consented to an assessment, especially as it was felt that many adults with intellectual disabilities do not have opportunities to be in control of their lives or to have positive relationships with social workers:

To actually be able to flex a muscle and make a choice and that choice be valid and respected, sometimes that in itself is the rationale …We need to ensure that they've consented, that they've got capacity, that they are in agreement with…We can't just chuck parent support at people (Site 5, focus group 2)

However, this is a complex area of practice, and it is important to understand why parents may refuse consent to be assessed or may not accept support. This is often conceptualised as ‘disengagement from services’. Previous social work involvement may have been experienced as negative or traumatic, particularly if the reason for involvement was around child protection. It may also be the case that the distinction between a Care Act assessment and an assessment of parenting capacity was not made clear. Indeed, participants recognised that ‘people are scared of social services’ (Site 4, focus group 2) as services were perceived as 'a danger' (Site 3, focus group 1), and this might put them off pursuing an assessment:

If they're scared, so obviously there's a stereotype around social care…I think that's something we get quite a lot, is the fear around what our job could do to their lives and things like that, so building those relationships can be quite challenging… (Site 4, focus group 2)

Participants did not, however, appear to fully appreciate the implications for parents if they refused a Care Act assessment, that is they might not get the support they need with their parenting, and therefore may not have discussed this fully with parents. It is therefore unclear if parents were able to make fully informed decisions on whether to consent to an assessment.

#### 3.2.2 Building Relationships: ‘Really Building That Relationship To Reduce Fear’

Recognising this fear of social services, some adult social workers tried to build relationships with parents prior to assessing their eligibility to receive support under the Care Act. They used many strategies to build these relationships such as visiting regularly and making their role clear and distinct from children's social workers. One worker explained how they would ask to accompany parents to meetings about their child so that they could ‘show’ direct support for the parent. These participants also avoided using the term ‘assessment’ due to the negative connotations discussed above, using less formal language to enable conversations about the parent's support needs. Building relationships and establishing trust takes time and this was particularly challenging in the current financial climate where severe resource constraints may act as a barrier to building relationships.

#### 3.2.3 Understanding and Application of the Care Act Eligibility Criteria: ‘it's Open to interpretation’

Although the Care Act eligibility criteria are set out clearly in *‘The Care and Support (Eligibility Criteria) Regulations 2015’* (UK Government 2015), they were regarded as being open to interpretation by individual workers:

The Care Act is…it's really thorough in some ways, but I do also think that it leaves a lot open to interpretation when you talk about people's wellbeing. (Site 1, focus group 3)

Managers from the general teams were particularly concerned about their teams' ability to assess whether a parent had an intellectual disability as well as *how* the parent's intellectual disability would impact on their capacity to parent. It was suggested that while general workers were skilled at carrying out assessments, they did not have the specialist knowledge, expertise, or tools to assess parenting capacity. This was particularly challenging because Care Act eligibility was based on whether a parent's mental or physical disability meant that they required support to carry out their parenting duties. Not all workers felt able to make this judgement. The assessment of these parents was felt to be somewhat different from the types of clients that general social workers routinely worked with, whom they referred to as their *‘bread and butter’*:

…the things that I would routinely do as an older person's social worker or the services I would ordinarily access many not be quite right for this person (Site 5, manager 2)

Managers and general social workers discussed two approaches to their application of the Care Act eligibility criteria. The first was very inclusive, which meant that most people could be viewed as meeting the eligibility criteria because of the perceived flexibility of the criteria. Social workers who adopted this inclusive approach assumed that if support was needed with parenting, then it was also highly likely that support would be required in other areas:

So, this would be, obviously, parenting and you'd find another one. Maybe social, or vocational … And then, you would say does that impact on their wellbeing by dint of those two things? And the answer will probably be ‘yes’ (Site 1, focus group 2)

These social workers *actively* looked for ways to ensure that it was possible for a parent without a diagnosed intellectual disability to receive support under the Care Act. The approach taken was characterised by openness, flexibility and a commitment to developing relationships.

On the other hand, there was a second group of participants who appeared to be much more ‘rule‐bound’ and conscious of resource constraints, which appeared to inform their practice. These participants were very focussed on eligibility criteria and believed that not all parents with milder or undiagnosed intellectual disabilities would have a second eligible care need, particularly if the parent had downplayed or minimised needs due to concerns about child removal or the perceived stigma of the assessment process. In cases where a parent had only one eligible care need, adult services would be unlikely to respond, and the parent would likely have input from children's services only, which operate very high thresholds, usually in relation to child protection concerns.

#### 3.2.4 Lack of Appropriate Knowledge and Skills: ‘we need to have really, really broad knowledge of everything and anything really… it's tricky’

As discussed above, participants in this study felt that having specialist knowledge of intellectual disability and parenting was important to ensure that parents got the most appropriately tailored support to meet their needs. Participants recognised that social workers in general teams had a wide range of knowledge, but this was usually around working with adults with a physical impairment or older people. They lacked specific experiences, skills and training around working with people with intellectual disabilities and found working with parents ‘challenging’ and complex:

People here that are allocated a parent with an intellectual disability are not going to have a scooby‐doo what they're doing, I think is the reality…there's only very few people that I think would feel really confident in the management of a case like that [a parent with an intellectual disability]. (Site 3, manager 1)

This manager noted that knowledge and understanding of intellectual disability were decreasing over time. Certain workers took on most of these cases and became viewed as ‘experts’ in working with these parents, resulting in the rest of the team feeling less confident. This reinforced concerns from managers of general teams that their teams were not the appropriate service to support this group of parents:

Anxiety is probably too strong a word, but a professional concern that are we the right service…that has access to the right sort of skilled people, the right training, the right experience, access to services that this person might possibly need? And so you can find… it builds to a tension point where… we're being dropped with these cases and we're not really the best, it really should be sent over there to the specialist team. (Site 5, manager 2)

### 3.3 The Invisibility of Parents With Intellectual Disabilities: Negotiating Thresholds in the Context of Austerity

It was noted that prior to the Care Act, many parents with intellectual disabilities would probably not have accessed support in their own right, ‘and they would have been going under the radar’ (Site 1, manager 1). Ten years after the implementation of the Act, participants suggested the number of parents with intellectual disabilities they worked with was still very small. This perception may mean that Local Authorities are less likely to commission services that might effectively meet these parents' needs. Indeed, managers and commissioners noted that the small numbers might mean that parents with intellectual disabilities ‘can fall off the agenda a little bit’ (Site 1, manager 3). This contributed to a sense that parents with intellectual disabilities were ‘a bit invisible’ (Site 3, manager 2).

Our findings have shown that parents with milder intellectual disabilities often occupy a ‘no‐man's land’ between intellectual disability and general adult services, characterised by uncertainty and invisibility. A great deal of effort was made by workers to manage the uncertainty and determine whether parents meet eligibility thresholds for services or not. This was uncomfortable work, compounded by austerity measures that have put increased pressure on resources:

We've got austerity and we've got reduced resources across the board, you know, every public service, I would say not one of them is…staffed to…the same level…and I think we are all trying to do much more with…much less. (Site 2, focus group 1)

This can contribute to a disconnect between policy and practice, whereby the context within which the policy is being operationalised is not taken sufficiently into account. This has led to workers becoming ‘very, very protectionist’ (Site 1, manager 2) in their approach:

You know it [the Care Act] wasn't launched with additional money…I think we would probably all acknowledge…the possibility and opportunity…that was envisaged in… the writing of the legislation possibly isn't quite the same reality under current circumstances. (Site 5, manager 3)

This has an impact on access to support for parents with intellectual disabilities, and there can be significant consequences and implications for the families in question:

These two parents ended up losing their children to adoption…but I often wonder, if the support had been there from the beginning…would things have turned out differently…? That…is my experience of the intellectual disabilities team. Their thresholds are so high that…you're probably going to struggle to…I'll be honest with you…I wouldn't even bother making a referral. (Site 1, focus group 1)

## 4 Discussion

The aim of this study was to investigate how adult social workers identified parents with intellectual disabilities and how services respond to them. To address the research questions set out above, we wished to explore how, when and from whom parents with intellectual disabilities accessed support. We found that this was an area of complexity and ambiguity, with difficulties around navigating the threshold for support under the Care Act. Given that NIHR (Health Research Funder in England) and the Chief Social Work Officer for Adults in England identified Care Act implementation as a priority area for social care research, the findings from this work are particularly timely and important.

Overall, we found that the term intellectual disability was often poorly understood, mirroring the existing literature on other professionals' experiences of working with this group or very rigidly applied to determine access to support. Both these positions required workers to make threshold judgements (Platt and Turney 2014; Turney et al. 2024). We found two clear entry points or thresholds into services where critical judgements are made by workers to determine whether parents meet eligibility criteria.

### 4.1 Threshold One

We found that the likely presence or absence of an intellectual disability typically determined whether an individual parent met the threshold to access support from the intellectual disability team. This judgement would usually be supported by a cognitive assessment, involving multi‐disciplinary colleagues such as psychologists and other allied health professionals, and other appropriate assessment tools. It was often based, in part, on IQ measurement, which has been largely critiqued for paying insufficient attention to contextual issues (Williams et al. 2022). McConnell et al. (2006) and Collings and Llewellyn (2012) further argue that IQ tests are a poor predictor of parenting ability and levels of need. Indeed, there appears to be a group of parents with intellectual disabilities who had needs that were too complex for general adult social care teams yet did not meet the eligibility criteria for the intellectual disability service because they did not meet the IQ threshold. They were effectively let down by the system.

The findings suggest that those parents with intellectual disabilities who can access specialist support from the intellectual disability team will receive more appropriately tailored support from workers with greater knowledge and understanding of intellectual disabilities and greater access to resources. Yet, our participants generally felt that the threshold to access support from these specialist teams was extremely high. According to Public Health England (2016), most adults with intellectual disabilities in England do not use intellectual disability services due to the operation of eligibility criteria to ration access to specialist support, and the stigma that leads to an unwillingness to self‐identify as having an intellectual disability. They refer to the ‘hidden majority’ (Hatton 2016) of adults with intellectual disabilities who remain unseen in data collection, much like those in our study.

### 4.2 Threshold Two

The second entry route or decision‐making threshold was for those parents not eligible for support from the intellectual disability team. In these cases, judgements centred on whether these parents were eligible to receive support under the Care Act. To make these decisions, workers in general adult teams felt they required greater levels of knowledge and understanding of intellectual disability and its likely impacts on parenting. This reflects the concerns and uncertainties expressed by workers in previous studies (Jones 2013; Tefre 2017, McConnell and Llewellyn, 2002; Pacheco and McConnell 2017; Tilbury and Tarleton 2023).

The Care Act appears to offer potential for those parents with eligible care needs to access support. However, while our study found evidence of workers using Care Act eligibility criteria flexibly and proactively to ensure parents had two eligible needs, there were also examples where eligibility criteria were used to restrict access to support. Our findings suggest that this was because the implementation of the Care Act brought no additional money or resources to LAs – despite substantially increasing the numbers of people who might come forward for assessment and be subsequently eligible for support (Fernandez et al. 2014). This exacerbated workers' uncertainty about making these assessments and their ability to provide appropriate support. Austerity has had a severe impact on social care budgets, and this has resulted in high levels of unmet need, with providers struggling to deliver quality care amidst a workforce crisis (Kings Fund 2022). The Care Quality Commission found that in 2021/22 over 205,000 adults aged 18 to 64 were not provided with the social care support they requested. Parents with milder or borderline intellectual disabilities who are invisible to adult services are surely part of this unserved group.

An additional complexity when considering threshold two is whether parents are willing to agree to a Care Act Assessment. Indeed, adult social workers' sometimes unquestioning acceptance of parents' right to refuse, possibly as part of their commitment to anti‐oppressive practice, potentially reduces the number of parents being assessed under the Care Act but can have major implications for their parenting and their involvement with children's services regarding the welfare of their children. Such parents are unlikely to receive support from adult services and are likely to be picked up further down the line by children's services. Burch et al.'s (2024) findings regarding the higher numbers of parents with intellectual disabilities involved in judicial proceedings appear to add weight to this argument.

Our findings suggest that practices within LAs alongside a lack of understanding around the nature of their needs may render these parents invisible, resulting in a failure to commission appropriate services to support them. Current supports available from general teams are often centred around traditional homecare services that are well placed to support older adults or people with physical disabilities but are likely to lack experience of, and registration for, family‐centred support.

Our findings suggest that the ‘invisibility’ of parents with mild or borderline intellectual disabilities might allow workers to make sense of the ways that access to support is restricted. The current financial climate and political context within which social workers are currently practising cannot be separated from this sense‐making process (Platt and Turney 2014). We argue that austerity measures mean that parents with intellectual disabilities *need* to be invisible, as there simply are not enough resources to support them. Indeed, if parents with intellectual disabilities remain “unseen” then they cannot be supported. This has led to resource‐led (rather than outcomes‐led) decision making, which is at odds with the preventative nature of the Care Act (2014).

### 4.3 Study Limitations

We had originally planned a study that included more participants from each site; however, we believe that, in addition to the time pressures on social workers, we were asking uncomfortable questions regarding social work practice about a group that they may not be supporting or that local systems and policies were not focused on. A further limitation of the study is its very specific focus on a particular legislative context (i.e., the Care Act as implemented in England). However, we believe the findings and key messages presented here have wider applicability across other jurisdictions and countries where the term intellectual disabilities is, at times, contested and misunderstood and where social care services are being placed under increasing demands and fiscal pressures.

## 5 Conclusion

Parents with milder or borderline intellectual disabilities are a group of parents who may not have been eligible for social care support prior to the Care Act 2014. While the Act appeared to have the potential to enable adult services to support this group of parents, the huge resource constraints currently facing adult services has meant the Care Act has not been operationalised as envisaged. Access to the person‐centred support provided by intellectual disability teams is protected by a strict IQ‐based eligibility criterion while access to support under the Care Act from general teams is protected by some social workers, hemmed in by their perceived lack of skills and the need to protect scarce resources. This is despite Article 23 of the UN Convention on the Rights of Persons with Disabilities stating that states are bound to ‘render appropriate assistance to persons with disabilities in the performance of their child‐rearing responsibilities’. While it is apparent that social workers are trying to do a great deal with less resources, rendering parents' support needs invisible goes some way to explaining the over‐representation of parents with intellectual disabilities in the child protection system (Burch et al. 2024; Llewellyn and Hindmarsh 2015), as parents may be left struggling in a ‘no‐man's land’ without the support they need.

## Ethics Statement

The study was approved by the School for Policy Studies Research Ethics committee at the University of Bristol and each Local Authority's research governance process. All of the participants provided fully informed consent which included permission to publish their anonymised quotations and to archive their data.

## Conflicts of Interest

The authors declare no conflicts of interest.

## Data Availability

The data that support the findings of this study are available on request from the corresponding author. The data are not publicly available due to privacy or ethical restrictions. The data has been archived at data.bris.ac.uk. Access is closed.
